# Knowledge of the Teeth among the Ancients

**Published:** 1856-01

**Authors:** 


*Knowledge of the Teeth among the Ancients.*—As a curious relic of ancient dental literature, we copy the following from Pliny's Natural History.—Eds.

It is a matter beyond doubt, that in young children the front teeth are produced at the seventh month, and, nearly always, those in the upper jaw first. These are shed in the seventh year, and are then replaced by others. Some infants are even born with teeth. Such was the case with Marius Curius, who, from this circumstance, received the name of Dentatus; and also with Cn. Papirius Carbo, both of them distinguished men. When this phenomenon happened in the case of a female, it was looked upon in the time of the kings, as an omen of some inauspicious event. At the birth of Valeria, under such circumstances as these, it was the answer of the soothsayers, that any city to which she might happen to be carried would be destroyed; she was sent to Suessa Pometia, at that time a very flourishing place, but the prediction was ultimately verified by its destruction. * * * Some persons are born with a continuous bone in their mouth, in the place of teeth; this was the case with the upper jaw of the son of Prusias, the king of Bithynia.

The teeth are the only parts of the body which resist the action of fire, and are not consumed along with the rest of it. Still, however, though they are able thus to resist flame, they become corroded by a morbid state of the saliva. The teeth are whitened by certain medicinal agents. They are worn down by use, and fail in some persons long before any other part of the body. They are necessary, not only for the mastication of the food, but for many other purposes. It is the office of the front teeth to regulate the voice and the speech; by a certain arrangement, they receive, as if in concert, the stroke communicated by the tongue, while by their structure in such regular order, and their size, they cut short, moderate, or soften the utterance of the words. When they are lost, the articulation becomes altogether confused and indistinct.

In addition to this, it is generally supposed that we may form prognostics from the teeth. The number of teeth allotted to all men, with the exception of the nation of the Turduli, is thirty-two; those persons who have a greater number, are thought to be destined to be long-lived. Women have fewer teeth than men. Those females who happen to have two canine teeth on the right side of the upper jaw, have promise of being the favorite of fortune, as was the case with Agrippina, the mother of Domitius Nero; when they are on the left side, it is just the contrary. It is the custom of most nations not to burn the bodies of children who die before they have cut their teeth.—*Vol. 2, book 7, chap. 15, p.* 153.

